# A PTG Variant Contributes to a Milder Phenotype in Lafora Disease

**DOI:** 10.1371/journal.pone.0021294

**Published:** 2011-06-30

**Authors:** Rosa Guerrero, Santiago Vernia, Raúl Sanz, Irene Abreu-Rodríguez, Carmen Almaraz, María García-Hoyos, Roberto Michelucci, Carlo Alberto Tassinari, Patrizia Riguzzi, Carlo Nobile, Pascual Sanz, José M. Serratosa, Pilar Gómez-Garre

**Affiliations:** 1 Laboratorio de Neurología-Unidad de Epilepsia, Servicio de Neurología, Instituto Investigación Sanitaria Fundación Jiménez Díaz, and Centro de Investigación Biomédica en Red de Enfermedades Raras (CIBERER), Madrid, Spain; 2 Instituto de Biomedicina de Valencia, Consejo Superior de Investigaciones Científicas (CSIC), and Centro de Investigación Biomédica en Red de Enfermedades Raras (CIBERER), Valencia, Spain; 3 Laboratorio de Investigaciones Biomédicas, Instituto de Biomedicina de Sevilla (IBiS), Sevilla, Spain; 4 Unit of Neurology, Department of Neurosciences, Bellaria Hospital, Bologna, Italy; 5 Section of Padua, CNR-Institute of Neurosciences, Padua, Italy; 6 Unidad de Trastornos del Movimiento, Servicio de Neurología y Neurofisiología Clínica, Instituto de Biomedicina de Sevilla (IBiS), Sevilla, Spain; 7 University of Bologna, Bologna, Italy; Instituto de Ciencia de Materiales de Madrid - Instituto de Biomedicina de Valencia, Spain

## Abstract

Lafora disease is an autosomal recessive form of progressive myoclonus epilepsy with no effective therapy. Although the outcome is always unfavorable, onset of symptoms and progression of the disease may vary. We aimed to identify modifier genes that may contribute to the clinical course of Lafora disease patients with *EPM2A* or *EPM2B* mutations. We established a list of 43 genes coding for proteins related to laforin/malin function and/or glycogen metabolism and tested common polymorphisms for possible associations with phenotypic differences using a collection of Lafora disease families. Genotype and haplotype analysis showed that *PPP1R3C* may be associated with a slow progression of the disease. The *PPP1R3C* gene encodes protein targeting to glycogen (PTG). Glycogen targeting subunits play a major role in recruiting type 1 protein phosphatase (PP1) to glycogen-enriched cell compartments and in increasing the specific activity of PP1 toward specific glycogenic substrates (glycogen synthase and glycogen phosphorylase). Here, we report a new mutation (c.746A>G, N249S) in the *PPP1R3C* gene that results in a decreased capacity to induce glycogen synthesis and a reduced interaction with glycogen phosphorylase and laforin, supporting a key role of this mutation in the glycogenic activity of PTG. This variant was found in one of two affected siblings of a Lafora disease family characterized by a remarkable mild course. Our findings suggest that variations in PTG may condition the course of Lafora disease and establish PTG as a potential target for pharmacogenetic and therapeutic approaches.

## Introduction

Lafora disease (LD; MIM#254780) is an autosomal recessive form of progressive myoclonus epilepsy that typically manifests during adolescence and is characterized by epilepsy, progressive neurologic deterioration, myoclonus and epileptic seizures. The disease leads to a vegetative state and death, usually within less than a decade from the onset of the initial symptoms [1]. The hallmark of LD is the presence of polyglucosan intracellular bodies, first described by Lafora and Glueck in 1911 [2]. These inclusions have been found in many tissues, including brain, spinal cord, liver, skin, skeletal muscle, heart and retina [3].

Two genes have been associated with LD: *EPM2A* [4]–[5] and *EPM2B* [6]. *EPM2A* (MIM#607566) is a four-exon gene which encodes a protein known as laforin. Initially, laforin was partially characterized and described as a dual-specificity phosphatase [4]–[5]. Subsequently, the complete coding human sequence of the gene including the ATG initiation codon region was reported [7]. The complete protein shows an amino-terminal carbohydrate binding module (CBM) that is critical for association with glycogen both *in vitro* and in *vivo* [8]. *EPM2B* (MIM#608072) is a single-exon gene which codes for an E3-ubiquitin ligase, known as malin, that contains a RING finger domain and six NHL-domains involved in protein-protein interactions [7], [9]. Malin interacts with and ubiquitinates laforin, leading to its degradation [9].

Laforin and malin appear to be part of a multiprotein complex that may be associated with the formation of intracellular glycogen particles. Within this complex, laforin interacts with protein targeting to glycogen known as PTG (Gene symbol *PPP1R3C*, MIM#602999), one of the glycogen targeting regulatory subunits of protein phoshatase 1 (PP1) [10]. Recently, it has been shown that the glycogenic activity of PTG is down-regulated by the laforin-malin complex by inducing protesome-dependent degradation [11]–[13]. However, the absence of malin in mice does not affect the levels of glycogen synthase, PTG or debranching enzyme [14].

Lafora disease patients show variability in the age and symptoms at onset, as well as in the duration of disease progression, even among affected siblings with the same mutation [15]–[19]. The striking finding of hepatic disease as the first manifestation of LD in one of two affected siblings, suggests that modifier genes must condition the clinical expression of the disease [20].

To determine if genes involved in the regulation of *EPM2A* and *EPM2B* can modify the onset and progression of the disease, we screened a total of 43 genes coding for proteins related to laforin/malin function and/or glycogen metabolism in a collection of LD families that showed intrafamilial phenotypic differences. We found two heterozygous variations in the *PPP1R3C* gene. In this work we tested the significance of these variations on the glycogenic capacity of human PTG and their possible role in an exceptionally mild form of the disease.

## Results

### Haplotype analysis reveals *PPP1R3C* as a candidate gene that modifies disease progression

We evaluated if LD patients with intrafamilial phenotypic differences (age at onset or disease progression) presented distinct haplotypes for each of the selected 43 gene *loci* related to laforin/malin function or to glycogen metabolism (*see* *Materials and Methods*).

Genotyping analysis showed a possible association of *PPP1R3C* with a slow progression of the disease. Since PTG, the gene product of *PPP1R3C*, has previously been shown to interact directly with laforin [10], we focused our attention on this gene.

### Analysis of *PPP1R3C* mutations

Sequencing analysis of the two exons, the promoter region and untranslated regions of *PPP1R3C* was carried out in a total of 23 LD patients.

**Figure 1:**
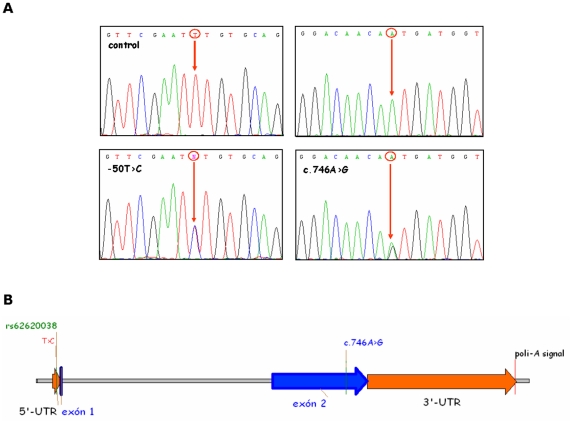
Mutational analysis of *PPP1R3C*. (A) Electropherograms show the two containing-variation regions in control (top) and affected individuals (bottom). Nucleotides that change are indicated. B) Schematic drawing of the genomic structure of *PPP1R3C*. Position of the variations found in Lafora disease patients is shown.

**Figure 2:**
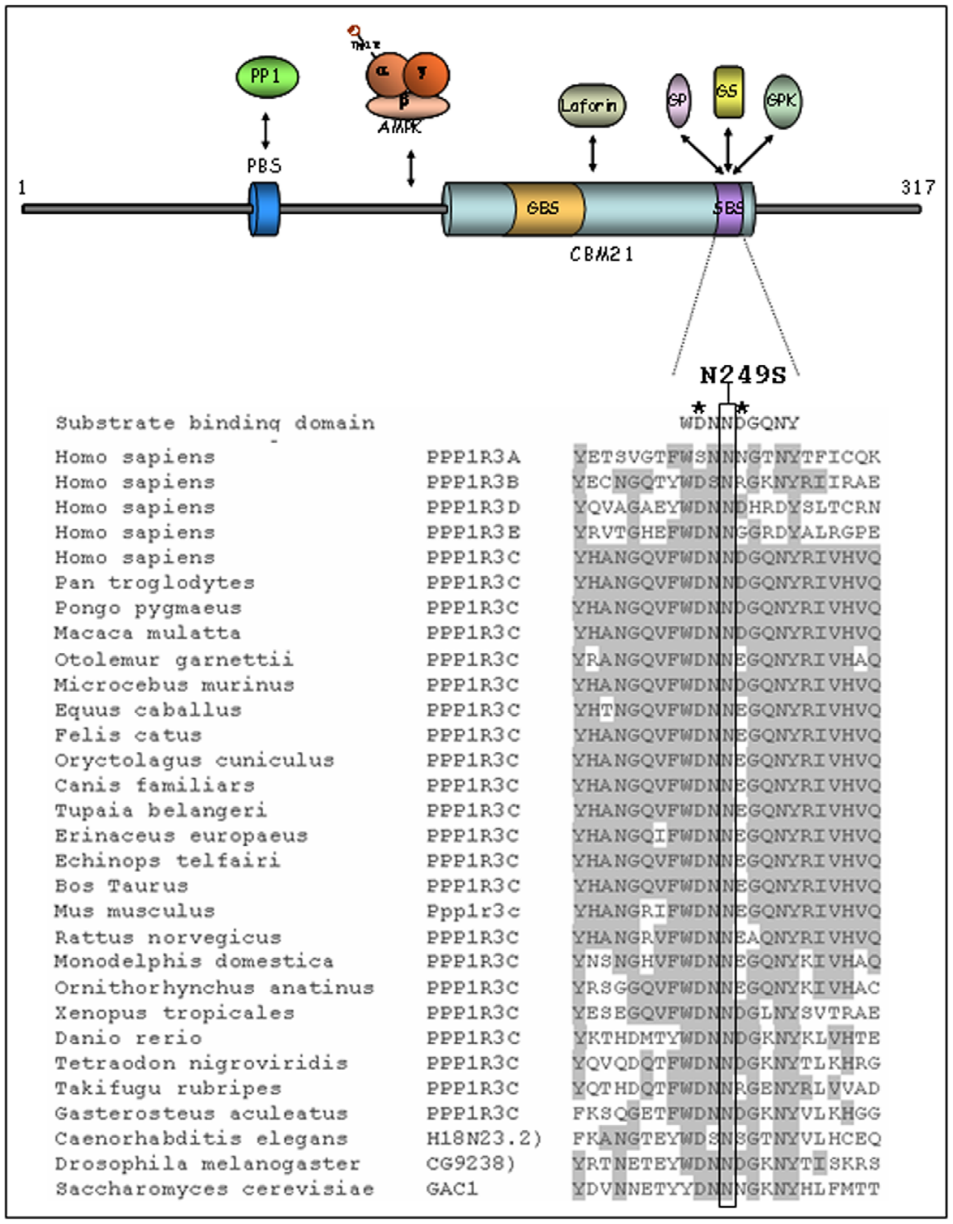
Schematic drawing of PTG domain structure and multiple alignments of human PTG amino acid sequence. Multiple alignments of the motif shared by substrate binding domain of human PTG amino acid sequence and paralogues and homologues from various species are shown and identities are shadowed. The mutated asparagine residue (position 249 in the protein) is indicated with a vertical box. Asterisks mark esential aspartic residues that surround asparagine. Abbreviations are as follows: PBS, PP1 binding site; CBM21, carbohydrate binding module type-21; GBS, glycogen binding site; SBS, substrate binding site; PP1, protein phosphatase 1; AMPK, AMP-activated protein kinase; GPh, glycogen phosphorylase; GS, glycogen synthase; GPK, glycogen phosphorylase kinase.

We identified two distinct heterozygous DNA transitions in our patients: one in the coding sequence and one in the 5′untranslated region of *PPP1R3C* (Figure 1). Variations were named based on mRNA reference sequence NM_005398 and protein sequence NP_005389. The variation in the coding region was an amino acid substitution of asparagine for serine at position 249 (**N249S**, c.746A>G). This residue is completely conserved throughout evolution (Figure 2). The other transition (**−50T>C**) was found in the promoter region and matched with a known SNP (rs62620038). No mutations segregating with the phenotype were identified in the 3′-untranslated region. The N249S variant was found in patient 127-3, who also carried two mutations in *EPM2B*, D146N and S339fsX351. The −50T>C variant was found in patient 111-3 who had a homozygous deletion in *EPM2A* (Ex1_33bpdel, R31_R41del).

Subsequent TaqMan probe assays were designed in order to test these variants in an additional sample of 30 LD affected individuals and in Spanish (n = 94) and Italian (n = 119) control populations. The −50T>C variant was present in eight control but c.746A>G (N249S) was absent in all the tested controls and in dbSNP.

### Clinical findings

***Individual 127-3*** is a 43 year old Italian woman. Although she had two isolated tonic-clonic seizures at ages 5 and 6 years, she was in good health until the age of 22, when she had a new tonic-clonic seizure and reported onset of myoclonic jerks. Since then, the neurological picture progressed slowly. An EEG and a skin biopsy were consistent with the diagnosis of LD. Presently, she has resting and action myoclonus, mental deterioration and cerebellar signs. However, she is still almost completely autonomous.

***Individual 111-3*** was a Spanish woman last seen at the age of 19 years with seizure onset at the age of 14 years, (generalyzed tonic-clonic seizure). She presented a severe evolution of the disease with absence seizures, myoclonic jerks and mental deterioration. Her EEG and skin biopsy were consistent with the diagnosis of LD.

### PTG carrying the N249S mutation shows a decreased interaction with some PTG interacting partners

**Figure 3:**
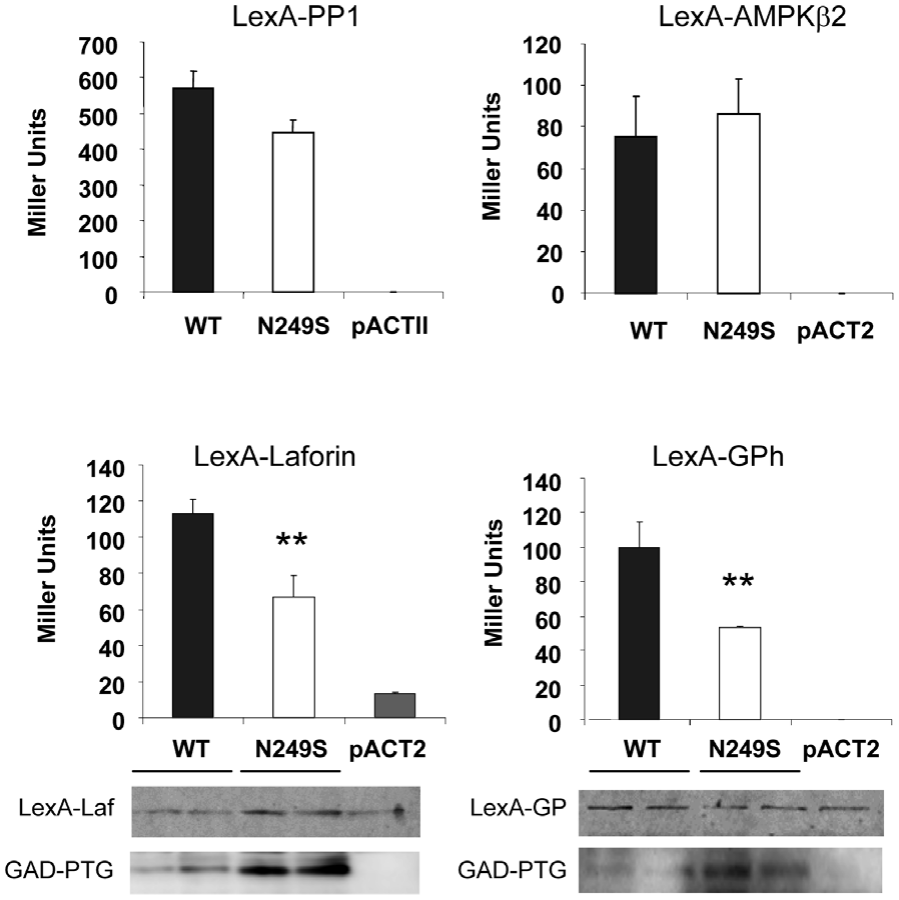
Yeast two-hybrid interaction assays of PTG with different proteins. Yeast CTY10.5d strain was transformed with plasmids pACT2 (empty plasmid), pACT2- PTG (wt), or pACT2-N249S-PTG (N249S) and co-transformed with plasmids pBTM-PP1α (LexA-PP1), pBTM-AMPKβ2 (LexA-AMPKß2), pEG202-laforin (LexA-laforin) and pEF202-RABPYGM (LexA-GPh). Protein interaction was estimated by measuring the β-Galactosidase activity in permeabilized yeast cells and expressed in Miller units. Abbreviations: PP1α, protein phosphatase 1; AMPKβ2, AMP-activated protein kinase subunit β2; GPh, glycogen phosphorylase. When indicated, crude extracts (25 µg) from two independent transformants were analyzed by western blotting using anti-LexA and ant-HA antibodies.

To check whether the N294S mutation affected the interaction of PTG with some of its partners (Figure 2), we performed a yeast two-hybrid analysis with protein phosphatase 1 (PP1), glycogen phosphorylase (GPh), AMP-activated protein kinase subunit β2 (AMPKβ2) and laforin. As shown in Figure 3, the N294S mutant form interacted with PP1 and AMPKß2 with a similar strength as the wild type. However, the interaction with laforin and glycogen phosphorylase was significantly reduced. Western blot analysis indicated that the observed reduction in interaction was not due to reduced levels of the N249S mutant (Figure 3).

### N249S reduces glycogen synthesis induction capacity of PTG

**Figure 4:**
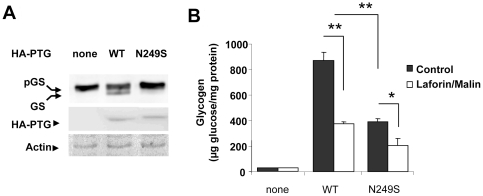
The presence of N249S in PTG reduces glycogen synthesis by blocking its capacity to activate GS. (A) Crude extracts from homogenates of cultured HEK293 cells transfected with pCMV-HA empty plasmid (none) or plasmids expressing wild type (WT) and mutated HA- PTG (N249S) were obtained and analyzed by Western blotting using anti-GS and anti- PTG. Actin was used as a control for gel loading. (B) HEK293 cells were co-transfected or not with plasmids pCMV-myc-laforin/pcDNA-HA-malin and with pCMV-HA (empty plasmid; none) or plasmids expressing wild type (WT) or mutated HA- PTG (N249S). Eighteen hours after the transfection, the amount of accumulated glycogen was measured as described in Materials and Methods.

In order to establish whether the N249S variation affects PTG function, we analyzed its capacity to modulate the PTG-induced accumulation of glycogen. Glycogen synthesis is a highly regulated process that depends on the activity of glycogen synthase (GS). This enzyme exists in two forms, an inactive extensively phosphorylated form (pGS) and an active dephosphorylated form. We aimed to determine whether the N249S-PTG mutant was active in dephosphorylating pGS. We transfected HEK293 cells with plasmids expressing wild type and the N249S mutant form of PTG and analyzed the phosphorylation status of GS by Western blotting. The expression of wild type PTG induced the appearance of dephosphorylated forms of GS (faster migrating bands), whereas the expression of the N249S mutant was not able to produce significant amounts of dephosphorylated GS (Figure 4A). We then analyzed the glycogenic properties of wild type and mutant forms. Consistent with previous reports [12], [21], [22] the expression of wild type PTG in HEK293 cells induced the accumulation of glycogen in these cells. However, the N249S-PTG mutant could only partially induce the accumulation of glycogen (Figure 4B). Moreover, we have recently described that the glycogenic activity of PTG is downregulated by the laforin-malin complex [12]. Consistent with this result, we observed a reduction of 57±2% in the glycogenic activity of wild type PTG when co-expressed with laforin and malin (Figure 4B). The glycogenic activity of the N249S-PTG mutant form was also reduced (44±9%) by the co-expression of laforin and malin (Figure 4B), indicating that this mutated form could still be a target of laforin-malin action. Perhaps this is the consequence of the partial reduction in the laforin- PTG interaction observed in the N249S mutant (44±8%; Fig. 3).

### N249S ameliorates the phenotype of the absence of laforin

**Figure 5:**
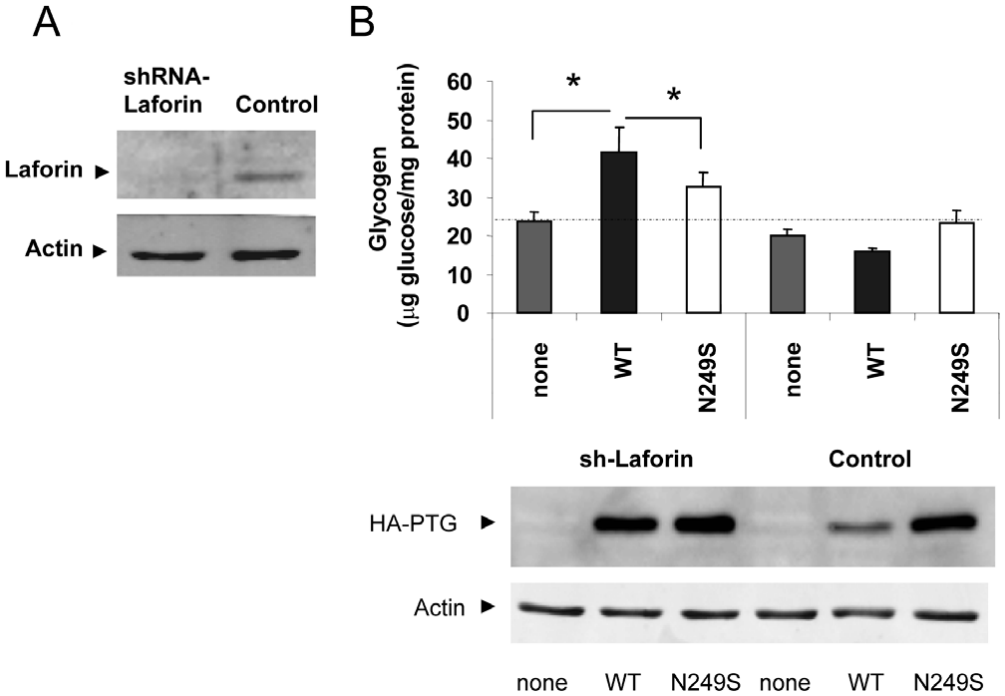
The N249S mutation in PTG diminishes the amount of glycogen accumulated in laforin-depleted cells. A) Human neuroblastoma SH-SY5Y cells were transfected with plasmid pSUPER-laf to deplete the levels of laforin. Crude extracts from these cells were analyzed by western blotting using anti-laforin and anti-actin antibodies. B) SH-SY5Y cells depleted (sh-laforin) or not (control) of laforin were transfected with plasmids pCMV-HA (empty plasmid; none) or plasmids expressing wild type (WT) or mutated HA- PTG (N249S). Eighteen hours after the transfection, the amount of accumulated glycogen was measured as described in *Materials and Methods*. The dotted line represent the levels of glycogen in control conditions. Crude extracts from the same cells were analyzed by western blotting using anti-HA and anti-actin antibodies.

To determine whether N249S-PTG mutant form could modify the glycogen accumulation phenotype in a neuronal cell line, we reduced the expression of laforin in a SH-SY5Y human neuroblastoma cells by shRNA (Figure 5A) and transfected these cells with either wild type or N249S-PTG mutant forms. We observed that only in the absence of laforin, was PTG wild type able to induce the accumulation of glycogen in these cells, indicating that in control cells laforin is a strong regulator of PTG activity (Figure 5B). In control cells we also observed a higher accumulation of the N249S mutant form, suggesting a lower capacity of the endogenous laforin to regulate the levels of this protein (Figure 5B). However, in laforin depleted cells, although the expression of N249S-PTG was similar to wild type, it promoted a lower accumulation of glycogen (Figure 5B). These results indicated that at least at the level of glycogen, the presence of the N249S variant ameliorated the phenotype produced by the absence of laforin.

### SNP rs62620038 influences gene reporter activity

**Figure 6:**
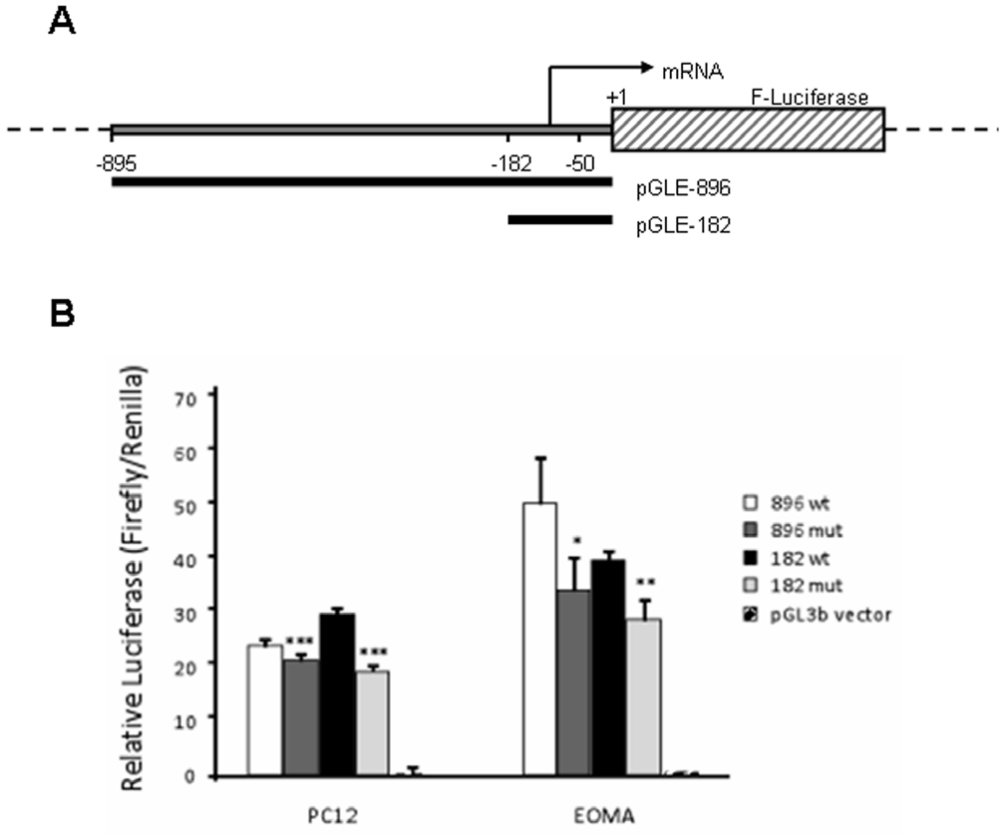
Impact of the −50T>C variant in pGL3 basic-derived constructs. A) Partial structures of constructs used in reporter assays. B) Gene reporter assays in PC12 and EOMA cells. Cells were transiently co-transfected with the reporter constructs containing the rs62620038 (position −50) variant or empty vector, as indicated. Each bar represents the average fold-induction relative to the empty vector.

The *PPP1R3C* 5′-UTR SNP has previously been denoted as rs62620038. However, there are no studies concerning the potential effect of this SNP on *PPP1R3C* expression. To analyze this point, an 896 bp 5′-UTR fragment was cloned into pGL3 luciferase reporter plasmid for transient transfection experiments in PC12 and EOMA cells (Figure 6A and 6B). Moreover, we constructed a 182 promoter fragment that was also analyzed (Figure 6A).

We observed that this short construct promoted the expression of the reporter gene at a similar extent as the longer promoter fragment, indicating that all regulatory elements of the *PPP1R3C* gene promoter were contained in the first 182 bp.

Mutated constructs carrying the −50T>C transition showed a statistically significant lower luciferase activity (24%) *vs* the corresponding wild type promoters (Figure 6B).

No statistically significant different promoter activity was conferred by constructs containing the −895 or −181 bp of the upstream sequence (Figure 6B). These results pointed out that the necessary positive regulatory elements for basal transcription reside in the most proximal region of the upstream sequence.

## Discussion

Lafora disease is an autosomal reccessive and fatal form of epilepsy characterized by the presence of cytoplasmatic aggregates of water-insoluble, poorly branched polyglucosans. These accumulate in the central and peripheral nervous system, among other tissues [23], [24]. However, it is unclear whether the accumulation of Lafora bodies is the cause of the disease or whether Lafora bodies are secondary determinants of a primarily established metabolic alteration [25]. Glycogen homeostasis depends mainly on the activity of enzymes involved in its synthesis (glycogen synthase or GS) and degradation (glycogen phosphorylase or GPh) through mechanisms involving phosphorylation [26], [27]. Type 1 protein phosphatase (PP1) dephosphorylates and activates GS and dephosphorylates and inactivates GPh and glycogen phosphorylase kinase (GPK) resulting in glycogen accumulation [26], [27]. The action of PP1 is controlled by several glycogen targeting subunits. PTG facilitates binding of PP1 to glycogen and acts as a molecular scaffold assembling PP1 with GS, GPh, and GPK at intracellular glycogen particles [11]. Thus, PTG modulates glycogen accumulation by bringing PP1 to GS for its activation [28].

Here, we report two PTG variants (one of them not previously described). One of these mutations replaces asparagine for serine at position 249 (N249S) and shows significant functional implications in glycogen metabolism. Asparagine 249 is a highly conserved amino acid located at the C-terminus of the protein and is surrounded by two aspartic acid residues that are essential for PTG activity since they are involved in the interaction of PTG with glycogenic substrates such as GS and GPh [27]. We provide evidence that this mutant form (N249S-PTG) results in a reduction of interaction with its partners GPh and laforin, and a decreased capacity to induce glycogen synthesis. In patients with *EPM2A* and *EPM2B* mutations loss-of-function of the laforin/malin complex results in PTG overexpression leading to the activation of GS and the accumulation of glycogen-associated PP1 [28]. The N249S-PTG mutant form has a lower glycogenic capacity and would counteract the up-regulation of glycogen synthesis observed in LD, possibly resulting in an amelioration of LD symptoms. We also provide evidence that this reduction in the glycogenic capacity of the N249S-PTG mutant form is due to its lower capacity to dephosphorylate and activate GS (Figure 4A).

The relevance of the functional studies presented here contrasts with the finding that only one of the affected siblings of family 127 carried the N249S variant in *PPP1R3C*. The patient who does not carry the mutation is, as his sib, almost completely autonomous but has clusters of diffuse myoclonic jerks on awakening. Both siblings carry the same mutations in *EPM2B* and mutations in this gene have been associated with a milder clinical course of the disease compared with *EPM2A* patients [18]. Moreover, it is remarkable that the patient with the PTG N249S mutation does not express other metabolic alterations in tissues where PTG is functional (liver, skeletal muscle, adipose tissue), indicating that other glycogen targeting subunits could compensate this deficiency.

Sequencing analysis of *PPP1R3C* in our patients revealed another variant located in the 5′-untranslated region that had been previously reported as a SNP (rs62620038). Deletion analysis in the upstream region of *PPP1R3C* showed that the elements responsible for basal promoter activity reside in the region proximal to the transcription initiation site, just where this SNP lies. Although this SNP was present in one LD patient and eight control individuals, luciferase reporter assays showed that this change affects human *PPP1R3C* promoter activity resulting in a mild decrease in gene expression. Perhaps, this may be the reason why the LD patient carrying this polymorphism does not present a modification in disease progression.

In conclusion, we identified a new variation in PTG that might contribute to ameliorate the symptoms of LD. However, other modifier factors may be involved in the clinical heterogeneity observed in Lafora disease. Our results suggest that PTG should be further studied as a potential pharmacogenetic target in LD.

## Materials and Methods

### Patients and samples

We studied 23 patients from 10 families diagnosed with LD. Diagnosis was based on the presence of epilepsy, myoclonus, rapidly progressive neurologic deterioration, and slow background with polyspike-wave complexes on EEG [1], [29]. A skin, muscle or liver biopsy was also required to confirm the presence of PAS-positive intracellular inclusions (Lafora bodies). Blood tests were reviewed when available in order to including cell blood count and complete biochemical panel to exclude liver, kidney or blood diseases.

All patients, except two, presented mutations in *EPM2A* or *EPM2B*.

Control subjects from Spanish and Italian populations were included in this study.

Blood was collected from patients and their relatives after informed consent. DNA samples were obtained from peripheral blood lymphocytes using standard methods. The study was approved by the Ethics Committee of the Fundación Jiménez Díaz.

### Genes selection and Genotyping

We selected 43 genes potentially involved in the regulation of laforin/malin function and/or glycogen metabolism. *EPM2A* and *EPM2B* were also included as control of association. An interaction network showing relationship among these genes is provided as supporting information (Figure S1). The network was performed using the online database resource Search Tool for the Retrieval of Interacting Genes (STRING, version 8.3). Interactions in STRING are provided with a confidence score and stronger associations are represented by thicker lines.

Genotyping was performed by analyzing polymorphic markers: 39 tagging single-nucleotide polymorphisms (tSNPs) from the HapMap database for population CEU and 95 microsatellites that included all gene *loci* linked to the selected genes. Haplotypes were determined by segregation analysis in families.

Common SNPs were chosen using Tagger-Pairwise algorithm with a r^2^>0.8 and a minor allele frequency (MAF)>0.2.

We assessed tSNPs by using TaqMan® SNP genotyping assays (Applied Biosystems, Foster City, CA, USA) and the 7000 Real-Time PCR System (Applied Biosystems). Genotyping success rate was 92.31% and the consensus rate was 100% (based on 100% duplicate genotypes).

Microsatellite sequences were amplified using PCR with a specific fluorescence- labelled primer. PCR products were run in an ABI Prism 3130 genetic analyzer (Applied Biosystems) and analyzed with the GeneMapper v3.5 software package (Applied Biosystems).

### Mutation analysis

*PPP1R3C* sequencing was performed from PCR-amplified genomic DNA fragments in both orientations. Sequence analysis covered all exonic sequences, exon/intron boundaries, and the regulatory 5′- and 3′-region and was performed by using dye terminator cycle sequencing kit (Applied Biosystems) with a ABI 3130 sequencer (Applied Biosystems).

### Cell culture and western blot

Human embryonic kidney (HEK293) and neuroblastoma (SH-SY5Y) cells were grown in DMEM (Lonza, Basel, Switzerland) and DMEM F12 (Lonza) respectively, supplemented with 100 units/ml penicillin, 100 µg/ml streptomycin, 2 mM glutamine (all from Sigma-Aldrich, USA) and 10% inactivated fetal bovine serum (FBS, GIBCO) at 37°C in an atmosphere of humidified 5% CO_2_. The day before transfection, 1.5×10^6^ cells were plated onto 60 mm-diameter culture dishes. Cells were transfected with 1 µg of each plasmid using Lipofectamine 2000 (Invitrogen-Life Technologies, Inc, Carlsbad, CA,USA). After twenty-four hours, the cells were scraped on ice in lysis buffer [10 mM TrisHCl pH 8; 150 mM NaCl, 15 mM EDTA; 0.6 M sucrose, 0.5% nonidet P-40 (NP-40), complete protease inhibitor cocktail (Roche Diagnostics, Meylan, France) and 1 mM PMSF, 50 mM NaF and 5 mM Na_2_P_2_O_7_] and lysed by repeated passage through a 25-gauge needle. Twenty-five micrograms of total protein, from the soluble fraction of cell lysates, were analyzed by SDS-PAGE and Western blotting using appropriate antibodies: mouse monoclonal anti-laforin [12], rabbit polyclonal anti- PTG (raised against the synthetic peptide (GPYDEFQRRHFVNK) corresponding to amino acids 16–29 of the human PTG) [28], mouse monoclonal anti-GS (Chemicon, Millipore, Billerica, MA, USA), mouse monoclonal anti-HA (Sigma) or rabbit polyclonal anti-actin (Sigma).

### Site-directed Mutagenesis

Site-directed mutagenesis of the PTG construct was performed using the QuikChange kit (Stratagene, La Jolla, CA, USA) and the following primers for **a) N249S**: forward, 5′- AATGGGCAAGTCTTTTGGGACAACAGTGATGGTCAGAATTATAGAATTGTT -3′, reverse, 5′- AATTCTATAATTCTGACCATCACTGTTGTCCCAAAAGACTTGCCCATT -3′, and for **b) 5′-UTR**: forward, 5′- CTGTGGTTCGAATCTGTGCAGGCAGCG-3′, reverse, 5′-CGCTGCCTGCACAGATTCGAACCACAG-3′.

Mutations and construct fidelity were confirmed by DNA sequencing.

### Plasmids

Plasmid pACT2- PTG contained the human full length PTG open reading frame [28]. Plasmid pACT2-PTG-N249S was generated by subcloning the mutated PTG fragment (described below) into pACT2 vector. A SfiI/BglII fragment from pACT2- PTG plasmid was subcloned into vector pCMV-HA (Clontech) in order to obtain wild type plasmid (pCMV-HA- PTG) and mutant plasmid (pCMV-HA-PTG-N249S).

Plasmid pEG202-RABPYGM (GPh), containing the rabbit full length muscular isoform of glycogen phosphorylase was obtained by subcloning a fragment from plasmid pEGFP-C1-MGP into pEG202. Other plasmids used in this study were pBTM-PP1α, pBTM-AMPKμ2, pEG202-laforin, pCMV-myc-laforin and pcDNA-HA-malin [30].

### Laforin siRNA silencing

The mammalian expression vector pSUPER.neo.GFP (Oligoengine) was used for expression of laforin-specific shRNA in human neuroblastoma SH-SY5Y cells. Sense and anti-sense oligonucleotides corresponding to nucleotides 872–891 of human EPM2A cDNA (GenBank accession no. NM_005670) were annealed and subcloned into pSUPER.neo.GFP vector, resulting in plasmid pSUPER-laf [30].

### Yeast two hybrid analysis

Yeast CTY10.5d strain was co-transformed with combinations of pACT2- PTG (wild type or N249S) and the indicated plasmids. Transformants were grown in selective SD medium and ß-galactosidase activity was assayed in permeabilized cells and expressed in Miller Units [12].

### Glycogen determination

Glycogen determination in transfected cells was carried out as described by Vernia *et al.* [28].

### Luciferase constructs

Analysis of *PPP1R3C* 5′-UTR was performed by insertion of a 890 pb fragment of the promoter region into vector pGL3 basic (Promega) using NcoI and HindII sites. This fragment was amplified by PCR using human genomic DNA as a template and appropriated primers. The 5′ oligo used was 5′-AAGCTTCTGCCAAAGGACGTCAGAATTG-3′ (HindII site underlined) and the 3′ oligo used was 5′-CCATGGTTAGGCAGAGAGGCGGCGGA-3′ (NcoI site underlined). The mutant plasmid was generated from the pGL3-896wt by site-directed mutagenesis. Plasmids with a 182 pb promoter were generated by digestion of pGL3-896wt and pGL3-896mut with XhoI and relegation and were subsequently denoted as pGL3-182wt and pGL3-182mut, respectively. The host strain for plasmid construction was *Escherichia coli* DH5α.

### Dual luciferase reporter assays

PC12 (derived from a pheochromocytoma of the rat adrenal medulla) and EOMA cells (derived from a mixed hemangioendothelioma present in an adult mouse) were cultured in 6-well plates and transfected at 50–60% confluence with 20 µg of individual reporter gene construct and *Renilla*/luciferase (100∶1 ratio) with Lipofectamine 2000 (Invitrogene). The total amount of DNA in each experiment was kept constant by using vector DNA controls. After forty-eight hours, cells were harvested in Passive Lysis Buffer (PLB, Promega), subjected to two freeze-thaw cycles and then precleared by centrifugation at 14,000×g for 1 min. Luciferase assays were performed in the supernatants using the Dual-Luciferase Reporter Assay System (Promega) according to the manufacturer's instructions. Luminiscence was measured with a Infinite 200 Nanoquant luminometer (Tecan Group, LTD). Values of firefly luciferase activity were normalized by those of *Renilla* luciferase activity.

### Statistical data analysis

Data are expressed as mean ± standard deviation (SD). Statistical evaluations were made by two-tailed Student's t test and one way ANOVA followed by the Student-Newman–Keuls multiple test as a *post-hoc* evaluation. Significance was ascribed at *p<0.05, **p<0.01 and *** p<0.001, as indicated in each case.

## Supporting Information

Figure S1 **Protein-protein interaction network visualized by STRING.** Confidence view of the network is shown for proteins potentially involved in the regulation of laforin/malin function and/or glycogen metabolism. Each protein is represented by circles. The color saturation of the edges represents the confidence score of the association. Stronger associations are represented by thicker lines.
(TIF)
