# Supplementary figures and images for: A PTG Variant Contributes to a Milder Phenotype in Lafora Disease
Source: PLoS One. 2011 Jun 30;6(6):e21294. doi: 10.1371/journal.pone.0021294 (PMC3127956; doi:10.1371/journal.pone.0021294)

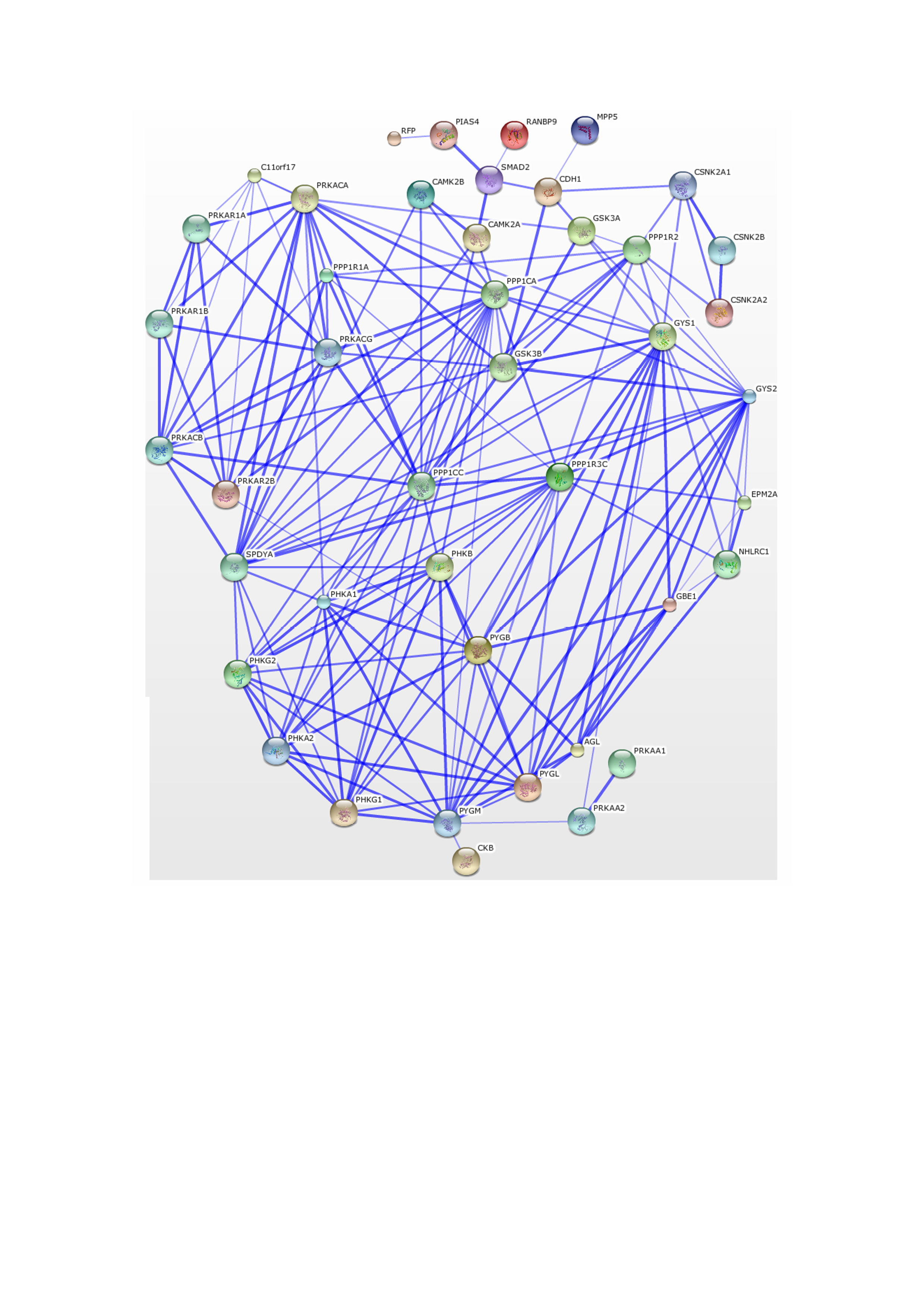

Supplement: Figure S1 — Protein-protein interaction network visualized by STRING. Confidence view of the network is shown for proteins potentially involved in the regulation of laforin/malin function and/or glycogen metabolism. Each protein is represented by circles. The color saturation of the edges represents the confidence score of the association. Stronger associations are represented by thicker lines. (TIF) [file pone.0021294.s001.tif]
